# 2,2,2-Trimethyl-*N*-(phenyl­sulfon­yl)­acetamide

**DOI:** 10.1107/S1600536808019983

**Published:** 2008-07-05

**Authors:** B. Thimme Gowda, Sabine Foro, B. P. Sowmya, P. G. Nirmala, Hartmut Fuess

**Affiliations:** a Department of Chemistry, Mangalore University, Mangalagangotri 574 199, Mangalore, India; b Institute of Materials Science, Darmstadt University of Technology, Petersenstrasse 23, D-64287 Darmstadt, Germany

## Abstract

The N—H and C=O bonds of the SO_2_—NH—CO group in the title compound, C_11_H_15_NO_3_S, are *anti* to each other. The asymmetric unit contains two independent mol­ecules. The benzene rings form dihedral angles of 83.19 (8) and 76.01 (10)° with the mean planes of the C_2_NOS fragments. The mol­ecules are linked into chains parallel to the *b* axis by inter­molecular N—H⋯O hydrogen bonds.

## Related literature

For related literature, see: Gowda, Nayak *et al.* (2007 ▶); Gowda, Foro & Fuess (2007 ▶); Gowda, Kožíšek *et al.* (2007 ▶); Gowda, Svoboda *et al.* (2007 ▶).
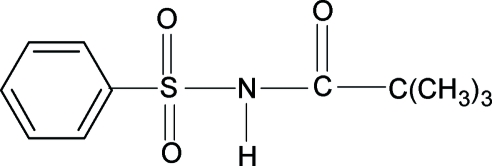


## Experimental

### 

#### Crystal data

C_11_H_15_NO_3_S
*M* *_r_* = 241.30
Monoclinic, 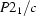

*a* = 12.3045 (9) Å
*b* = 11.3016 (7) Å
*c* = 18.466 (1) Å
β = 103.117 (6)°
*V* = 2500.9 (3) Å^3^
*Z* = 8
Mo *K*α radiation
μ = 0.25 mm^−1^
*T* = 299 (2) K
0.50 × 0.48 × 0.40 mm

#### Data collection

Oxford Diffraction Xcalibur diffractometer with a Sapphire CCD detector
Absorption correction: multi-scan (*CrysAlis RED*; Oxford Diffraction, 2007 ▶) *T*_min_ = 0.885, *T*_max_ = 0.906
15589 measured reflections
4985 independent reflections
3639 reflections with *I* > 2σ(*I*)
*R*_int_ = 0.029

#### Refinement

*R*[*F* ^2^ > 2σ(*F* ^2^)] = 0.051
*wR*(*F* ^2^) = 0.167
*S* = 1.16
4985 reflections
290 parameters
H-atom parameters constrained
Δρ_max_ = 0.44 e Å^−3^
Δρ_min_ = −0.40 e Å^−3^

### 

Data collection: *CrysAlis CCD* (Oxford Diffraction, 2007 ▶); cell refinement: *CrysAlis RED* (Oxford Diffraction, 2007 ▶); data reduction: *CrysAlis RED*; program(s) used to solve structure: *SHELXS97* (Sheldrick, 2008 ▶); program(s) used to refine structure: *SHELXL97* (Sheldrick, 2008 ▶); molecular graphics: *PLATON* (Spek, 2003 ▶); software used to prepare material for publication: *SHELXL97*.

## Supplementary Material

Crystal structure: contains datablocks I, global. DOI: 10.1107/S1600536808019983/rz2222sup1.cif

Structure factors: contains datablocks I. DOI: 10.1107/S1600536808019983/rz2222Isup2.hkl

Additional supplementary materials:  crystallographic information; 3D view; checkCIF report

## Figures and Tables

**Table 1:** Hydrogen-bond geometry (Å, °)

*D*—H⋯*A*	*D*—H	H⋯*A*	*D*⋯*A*	*D*—H⋯*A*
N1—H1N⋯O4^i^	0.86	2.09	2.946 (3)	171
N2—H2N⋯O2^ii^	0.86	2.32	3.094 (3)	151

Symmetry codes: (i) 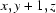; (ii) 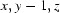.

**Fig. 1.**
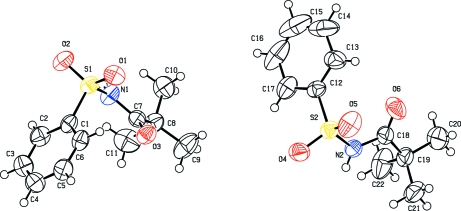
The molecular structure of the title compound, showing the atom labeling scheme and displacement ellipsoids drawn at the 50% probability level. H atoms are represented as small spheres of arbitrary radii.

**Fig. 2.**
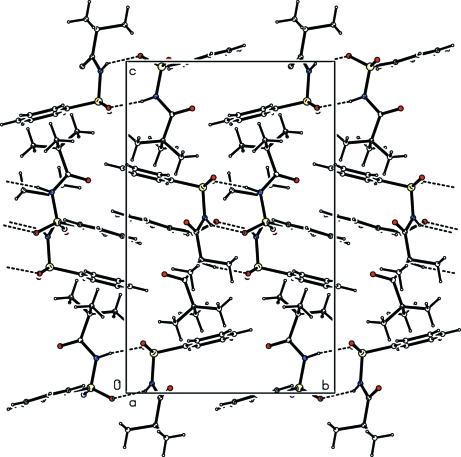
The molecular packing of the title compound viewed along tha a axis. Hydrogen bonds are shown as dashed lines.

## supplementary crystallographic information

### Comment

In the present work, as part of a study of the substituent effects on the solid state geometries of *N*-(aryl)-sulfonamides and substituted amides, the structure of *N*-(phenylsulfonyl)-2,2,2-trimethylacetamide (NPSTMAA) has been determined. The conformations of the N—H and C=O bonds of the SO_2_—NH—CO group in NPSTMAA are anti to each other (Fig. 1). The asymmetric unit of the structure contains two molecules. The bond parameters in NPSTMAA are similar to those in *N*-(aryl)-2,2,2-trimethylacetamides (Gowda, Foro & Fuess, 2007; Gowda, Kožíšek *et al.*, 2007; Gowda, Svoboda *et al.*, 2007) and benzenesulfonamide (Gowda, Nayak *et al.*, 2007). The benzene rings form dihedral angles of 83.19 (8) and 76.01 (10)° with the mean planes of the C_2_NOS fragments. A packing diagram of NPSTMAA molecules showing the formation of molecular chains parallel to the *b* axis through N—H···O hydrogen bonds (Table 1) is shown in Fig. 2.

### Experimental

The title compound was prepared by refluxing benzenesulfonamide (0.10 mol) in excess pivalyl chloride (0.20 mol) for about an hour on a water bath. The reaction mixture was cooled and poured into ice-cold water. The resulting solid was separated, washed thoroughly with water and dissolved in a warm sodium hydrogen carbonate solution. The title compound was precipitated by acidifying the filtered solution with glacial acetic acid. It was filtered, dried and recrystallized from ethanol. The purity of the compound was checked by determining its melting point. It was characterized by recording its infrared and NMR spectra. Single crystals of the title compound suitable for X-ray diffraction studies were obtained by slow evaporation of an ethanol solution.

### Refinement

The H atoms were positioned with idealized geometry using a riding model with C—H = 0.93–0.96 Å, N—H = 0.86 Å, and were refined with isotropic displacement parameters set to 1.2 times of the *U*_eq_ of the parent atom.

### Crystal data

**Table:**

C_11_H_15_NO_3_S	*F*_000_ = 1024
*M**_r_* = 241.30	*D*_x_ = 1.282 Mg m^−^^3^
Monoclinic, *P*2_1_/*c*	Mo *K*α radiation λ = 0.71073 Å
Hall symbol: -P 2ybc	Cell parameters from 7426 reflections
*a* = 12.3045 (9) Å	θ = 2.5–28.0º
*b* = 11.3016 (7) Å	µ = 0.25 mm^−^^1^
*c* = 18.466 (1) Å	*T* = 299 (2) K
β = 103.117 (6)º	Prism, colourless
*V* = 2500.9 (3) Å^3^	0.50 × 0.48 × 0.40 mm
*Z* = 8	

### Data collection

**Table:**

Oxford Diffraction Xcalibur diffractometer with a Sapphire CCD detector	4985 independent reflections
Radiation source: fine-focus sealed tube	3639 reflections with *I* > 2σ(*I*)
Monochromator: graphite	*R*_int_ = 0.029
*T* = 299(2) K	θ_max_ = 26.4º
Rotation method data acquisition using ω and φ scans	θ_min_ = 2.5º
Absorption correction: multi-scan(CrysAlis RED; Oxford Diffraction, 2007)	*h* = −15→15
*T*_min_ = 0.885, *T*_max_ = 0.906	*k* = −13→13
15589 measured reflections	*l* = −21→22

### Refinement

**Table:**

Refinement on *F*^2^	Hydrogen site location: inferred from neighbouring sites
Least-squares matrix: full	H-atom parameters constrained
*R*[*F*^2^ > 2σ(*F*^2^)] = 0.051	*w* = 1/[σ^2^(*F*_o_^2^) + (0.0676*P*)^2^ + 1.8991*P*] where *P* = (*F*_o_^2^ + 2*F*_c_^2^)/3
*wR*(*F*^2^) = 0.167	(Δ/σ)_max_ = 0.002
*S* = 1.16	Δρ_max_ = 0.44 e Å^−^^3^
4985 reflections	Δρ_min_ = −0.40 e Å^−^^3^
290 parameters	Extinction correction: SHELXL97 (Sheldrick, 2008), Fc^*^=kFc[1+0.001xFc^2^λ^3^/sin(2θ)]^-1/4^
Primary atom site location: structure-invariant direct methods	Extinction coefficient: 0.100 (4)
Secondary atom site location: difference Fourier map	

### Special details

**Table:**

Geometry. All e.s.d.'s (except the e.s.d. in the dihedral angle between two l.s. planes) are estimated using the full covariance matrix. The cell e.s.d.'s are taken into account individually in the estimation of e.s.d.'s in distances, angles and torsion angles; correlations between e.s.d.'s in cell parameters are only used when they are defined by crystal symmetry. An approximate (isotropic) treatment of cell e.s.d.'s is used for estimating e.s.d.'s involving l.s. planes.
Refinement. Refinement of *F*^2^ against ALL reflections. The weighted *R*-factor *wR* and goodness of fit *S* are based on *F*^2^, conventional *R*-factors *R* are based on *F*, with *F* set to zero for negative *F*^2^. The threshold expression of *F*^2^ > σ(*F*^2^) is used only for calculating *R*-factors(gt) *etc*. and is not relevant to the choice of reflections for refinement. *R*-factors based on *F*^2^ are statistically about twice as large as those based on *F*, and *R*- factors based on ALL data will be even larger.

### Fractional atomic coordinates and isotropic or equivalent isotropic displacement parameters (Å^2^)

**Table:**

	*x*	*y*	*z*	*U*_iso_*/*U*_eq_	
C1	0.6562 (2)	0.7079 (2)	0.00179 (14)	0.0448 (6)	
C2	0.7702 (3)	0.7222 (3)	0.00846 (18)	0.0580 (8)	
H2	0.8043	0.7948	0.0223	0.070*	
C3	0.8318 (3)	0.6267 (3)	−0.0058 (2)	0.0693 (9)	
H3	0.9082	0.6350	−0.0015	0.083*	
C4	0.7817 (3)	0.5195 (3)	−0.02624 (19)	0.0679 (9)	
H4	0.8244	0.4554	−0.0350	0.081*	
C5	0.6688 (3)	0.5067 (3)	−0.03380 (18)	0.0624 (8)	
H5	0.6350	0.4344	−0.0486	0.075*	
C6	0.6053 (3)	0.6003 (3)	−0.01956 (16)	0.0515 (7)	
H6	0.5289	0.5913	−0.0242	0.062*	
C7	0.5886 (2)	0.7797 (2)	0.15920 (15)	0.0440 (6)	
C8	0.6204 (3)	0.8245 (3)	0.23927 (15)	0.0541 (7)	
C9	0.5795 (4)	0.7357 (4)	0.2886 (2)	0.0894 (13)	
H9A	0.6138	0.6603	0.2849	0.107*	
H9B	0.5000	0.7281	0.2730	0.107*	
H9C	0.5990	0.7625	0.3393	0.107*	
C10	0.5687 (3)	0.9456 (3)	0.24601 (19)	0.0713 (9)	
H10A	0.4889	0.9399	0.2307	0.086*	
H10B	0.5953	1.0013	0.2148	0.086*	
H10C	0.5892	0.9718	0.2967	0.086*	
C11	0.7482 (3)	0.8339 (4)	0.2623 (2)	0.0856 (12)	
H11A	0.7740	0.8874	0.2295	0.103*	
H11B	0.7805	0.7572	0.2596	0.103*	
H11C	0.7698	0.8630	0.3124	0.103*	
N1	0.6084 (2)	0.8572 (2)	0.10554 (12)	0.0499 (6)	
H1N	0.6401	0.9236	0.1200	0.060*	
O1	0.46068 (17)	0.79593 (19)	−0.00518 (12)	0.0590 (6)	
O2	0.6126 (2)	0.93176 (18)	−0.01691 (11)	0.0613 (6)	
O3	0.54943 (18)	0.68328 (17)	0.14170 (11)	0.0541 (5)	
S1	0.57470 (6)	0.82976 (6)	0.01579 (4)	0.0467 (2)	
C12	0.8299 (2)	0.2840 (2)	0.14176 (14)	0.0476 (6)	
C13	0.9320 (3)	0.3399 (3)	0.15839 (18)	0.0678 (9)	
H13	0.9972	0.2991	0.1572	0.081*	
C14	0.9346 (6)	0.4580 (5)	0.1769 (2)	0.1085 (19)	
H14	1.0028	0.4973	0.1888	0.130*	
C15	0.8403 (8)	0.5176 (4)	0.1781 (3)	0.120 (2)	
H15	0.8442	0.5975	0.1905	0.144*	
C16	0.7397 (5)	0.4626 (4)	0.1615 (2)	0.0985 (16)	
H16	0.6752	0.5051	0.1625	0.118*	
C17	0.7324 (3)	0.3431 (3)	0.14311 (17)	0.0657 (9)	
H17	0.6640	0.3042	0.1321	0.079*	
C18	0.8663 (2)	0.1631 (3)	−0.01406 (16)	0.0531 (7)	
C19	0.8292 (3)	0.1401 (3)	−0.09727 (16)	0.0547 (7)	
C20	0.9144 (4)	0.1923 (4)	−0.1351 (2)	0.0894 (13)	
H20A	0.9856	0.1557	−0.1160	0.107*	
H20B	0.9202	0.2759	−0.1258	0.107*	
H20C	0.8918	0.1785	−0.1877	0.107*	
C21	0.8234 (4)	0.0072 (4)	−0.1109 (2)	0.0822 (11)	
H21A	0.7707	−0.0273	−0.0859	0.099*	
H21B	0.8957	−0.0271	−0.0921	0.099*	
H21C	0.8002	−0.0079	−0.1633	0.099*	
C22	0.7149 (4)	0.1925 (5)	−0.1277 (2)	0.0991 (15)	
H22A	0.7175	0.2764	−0.1193	0.119*	
H22B	0.6622	0.1573	−0.1030	0.119*	
H22C	0.6925	0.1771	−0.1801	0.119*	
N2	0.7948 (2)	0.1235 (2)	0.02886 (12)	0.0541 (6)	
H2N	0.7323	0.0925	0.0067	0.065*	
O4	0.7261 (2)	0.0845 (2)	0.13953 (12)	0.0719 (7)	
O5	0.9285 (2)	0.0813 (2)	0.15009 (14)	0.0811 (8)	
O6	0.9519 (2)	0.2109 (3)	0.01479 (13)	0.0823 (8)	
S2	0.82357 (6)	0.13285 (7)	0.12026 (4)	0.0512 (2)	

### Atomic displacement parameters (Å^2^)

**Table:**

	*U*^11^	*U*^22^	*U*^33^	*U*^12^	*U*^13^	*U*^23^
C1	0.0553 (15)	0.0441 (14)	0.0354 (12)	−0.0074 (12)	0.0111 (11)	−0.0005 (10)
C2	0.0561 (17)	0.0525 (17)	0.0625 (18)	−0.0116 (14)	0.0070 (14)	0.0000 (14)
C3	0.0572 (19)	0.076 (2)	0.077 (2)	0.0004 (17)	0.0198 (16)	0.0044 (18)
C4	0.081 (2)	0.061 (2)	0.068 (2)	0.0118 (17)	0.0315 (17)	−0.0010 (16)
C5	0.082 (2)	0.0481 (17)	0.0640 (19)	−0.0070 (15)	0.0306 (16)	−0.0093 (14)
C6	0.0598 (17)	0.0494 (16)	0.0481 (15)	−0.0122 (13)	0.0184 (12)	−0.0038 (12)
C7	0.0478 (14)	0.0402 (14)	0.0453 (14)	0.0023 (11)	0.0132 (11)	−0.0035 (11)
C8	0.0719 (19)	0.0524 (16)	0.0370 (14)	−0.0026 (14)	0.0103 (13)	−0.0038 (12)
C9	0.144 (4)	0.079 (3)	0.0471 (18)	−0.019 (3)	0.027 (2)	0.0023 (17)
C10	0.092 (3)	0.067 (2)	0.0593 (19)	0.0040 (19)	0.0272 (18)	−0.0160 (16)
C11	0.082 (3)	0.099 (3)	0.064 (2)	0.008 (2)	−0.0068 (19)	−0.011 (2)
N1	0.0684 (15)	0.0436 (12)	0.0376 (11)	−0.0118 (11)	0.0119 (10)	−0.0049 (9)
O1	0.0524 (12)	0.0616 (13)	0.0582 (12)	0.0000 (10)	0.0025 (9)	−0.0063 (10)
O2	0.0892 (16)	0.0466 (11)	0.0499 (11)	−0.0053 (11)	0.0195 (10)	0.0045 (9)
O3	0.0697 (13)	0.0420 (11)	0.0520 (11)	−0.0059 (9)	0.0165 (9)	−0.0025 (8)
S1	0.0585 (4)	0.0419 (4)	0.0387 (4)	−0.0035 (3)	0.0088 (3)	−0.0003 (3)
C12	0.0596 (17)	0.0470 (15)	0.0345 (13)	−0.0036 (12)	0.0071 (11)	−0.0018 (11)
C13	0.077 (2)	0.075 (2)	0.0491 (17)	−0.0233 (18)	0.0108 (15)	−0.0077 (15)
C14	0.172 (5)	0.084 (3)	0.068 (3)	−0.066 (4)	0.023 (3)	−0.021 (2)
C15	0.244 (8)	0.054 (3)	0.064 (3)	−0.014 (4)	0.039 (4)	−0.011 (2)
C16	0.164 (5)	0.078 (3)	0.057 (2)	0.056 (3)	0.031 (3)	0.005 (2)
C17	0.077 (2)	0.072 (2)	0.0467 (16)	0.0168 (17)	0.0098 (15)	0.0002 (15)
C18	0.0506 (16)	0.0640 (18)	0.0474 (16)	−0.0065 (14)	0.0164 (12)	−0.0095 (13)
C19	0.0563 (17)	0.0657 (19)	0.0445 (15)	−0.0009 (14)	0.0164 (12)	−0.0089 (13)
C20	0.111 (3)	0.112 (3)	0.053 (2)	−0.033 (3)	0.036 (2)	−0.012 (2)
C21	0.114 (3)	0.074 (2)	0.068 (2)	−0.010 (2)	0.040 (2)	−0.0204 (18)
C22	0.085 (3)	0.152 (4)	0.056 (2)	0.037 (3)	0.0070 (19)	−0.008 (2)
N2	0.0570 (14)	0.0692 (16)	0.0382 (12)	−0.0159 (12)	0.0152 (10)	−0.0152 (11)
O4	0.0986 (18)	0.0684 (15)	0.0565 (13)	−0.0316 (13)	0.0338 (12)	−0.0093 (11)
O5	0.0916 (18)	0.0805 (17)	0.0672 (15)	0.0328 (14)	0.0099 (13)	0.0079 (13)
O6	0.0625 (14)	0.132 (2)	0.0541 (13)	−0.0361 (15)	0.0165 (11)	−0.0164 (14)
S2	0.0652 (5)	0.0477 (4)	0.0410 (4)	−0.0019 (3)	0.0130 (3)	−0.0016 (3)

### Geometric parameters (Å, °)

**Table:**

C1—C6	1.384 (4)	C12—C13	1.377 (4)
C1—C2	1.389 (4)	C12—C17	1.378 (4)
C1—S1	1.757 (3)	C12—S2	1.752 (3)
C2—C3	1.378 (5)	C13—C14	1.376 (6)
C2—H2	0.9300	C13—H13	0.9300
C3—C4	1.373 (5)	C14—C15	1.347 (8)
C3—H3	0.9300	C14—H14	0.9300
C4—C5	1.372 (5)	C15—C16	1.357 (8)
C4—H4	0.9300	C15—H15	0.9300
C5—C6	1.375 (4)	C16—C17	1.391 (6)
C5—H5	0.9300	C16—H16	0.9300
C6—H6	0.9300	C17—H17	0.9300
C7—O3	1.206 (3)	C18—O6	1.195 (4)
C7—N1	1.384 (3)	C18—N2	1.386 (4)
C7—C8	1.527 (4)	C18—C19	1.523 (4)
C8—C9	1.516 (5)	C19—C20	1.506 (5)
C8—C10	1.526 (5)	C19—C22	1.512 (5)
C8—C11	1.536 (5)	C19—C21	1.522 (5)
C9—H9A	0.9600	C20—H20A	0.9600
C9—H9B	0.9600	C20—H20B	0.9600
C9—H9C	0.9600	C20—H20C	0.9600
C10—H10A	0.9600	C21—H21A	0.9600
C10—H10B	0.9600	C21—H21B	0.9600
C10—H10C	0.9600	C21—H21C	0.9600
C11—H11A	0.9600	C22—H22A	0.9600
C11—H11B	0.9600	C22—H22B	0.9600
C11—H11C	0.9600	C22—H22C	0.9600
N1—S1	1.644 (2)	N2—S2	1.647 (2)
N1—H1N	0.8600	N2—H2N	0.8600
O1—S1	1.421 (2)	O4—S2	1.434 (2)
O2—S1	1.428 (2)	O5—S2	1.410 (2)
			
C6—C1—C2	120.7 (3)	C13—C12—C17	121.9 (3)
C6—C1—S1	119.6 (2)	C13—C12—S2	119.3 (3)
C2—C1—S1	119.6 (2)	C17—C12—S2	118.8 (2)
C3—C2—C1	118.7 (3)	C14—C13—C12	118.0 (4)
C3—C2—H2	120.7	C14—C13—H13	121.0
C1—C2—H2	120.7	C12—C13—H13	121.0
C4—C3—C2	120.8 (3)	C15—C14—C13	121.2 (5)
C4—C3—H3	119.6	C15—C14—H14	119.4
C2—C3—H3	119.6	C13—C14—H14	119.4
C5—C4—C3	120.1 (3)	C14—C15—C16	120.9 (4)
C5—C4—H4	119.9	C14—C15—H15	119.6
C3—C4—H4	119.9	C16—C15—H15	119.6
C4—C5—C6	120.4 (3)	C15—C16—C17	120.3 (5)
C4—C5—H5	119.8	C15—C16—H16	119.9
C6—C5—H5	119.8	C17—C16—H16	119.9
C5—C6—C1	119.4 (3)	C12—C17—C16	117.8 (4)
C5—C6—H6	120.3	C12—C17—H17	121.1
C1—C6—H6	120.3	C16—C17—H17	121.1
O3—C7—N1	120.3 (2)	O6—C18—N2	120.0 (3)
O3—C7—C8	123.9 (3)	O6—C18—C19	124.0 (3)
N1—C7—C8	115.8 (2)	N2—C18—C19	116.0 (2)
C9—C8—C10	110.1 (3)	C20—C19—C22	111.2 (4)
C9—C8—C7	108.4 (3)	C20—C19—C21	108.7 (3)
C10—C8—C7	110.9 (2)	C22—C19—C21	108.7 (3)
C9—C8—C11	109.7 (3)	C20—C19—C18	108.7 (3)
C10—C8—C11	109.6 (3)	C22—C19—C18	110.4 (3)
C7—C8—C11	108.2 (3)	C21—C19—C18	109.1 (3)
C8—C9—H9A	109.5	C19—C20—H20A	109.5
C8—C9—H9B	109.5	C19—C20—H20B	109.5
H9A—C9—H9B	109.5	H20A—C20—H20B	109.5
C8—C9—H9C	109.5	C19—C20—H20C	109.5
H9A—C9—H9C	109.5	H20A—C20—H20C	109.5
H9B—C9—H9C	109.5	H20B—C20—H20C	109.5
C8—C10—H10A	109.5	C19—C21—H21A	109.5
C8—C10—H10B	109.5	C19—C21—H21B	109.5
H10A—C10—H10B	109.5	H21A—C21—H21B	109.5
C8—C10—H10C	109.5	C19—C21—H21C	109.5
H10A—C10—H10C	109.5	H21A—C21—H21C	109.5
H10B—C10—H10C	109.5	H21B—C21—H21C	109.5
C8—C11—H11A	109.5	C19—C22—H22A	109.5
C8—C11—H11B	109.5	C19—C22—H22B	109.5
H11A—C11—H11B	109.5	H22A—C22—H22B	109.5
C8—C11—H11C	109.5	C19—C22—H22C	109.5
H11A—C11—H11C	109.5	H22A—C22—H22C	109.5
H11B—C11—H11C	109.5	H22B—C22—H22C	109.5
C7—N1—S1	123.86 (19)	C18—N2—S2	123.3 (2)
C7—N1—H1N	118.1	C18—N2—H2N	118.4
S1—N1—H1N	118.1	S2—N2—H2N	118.4
O1—S1—O2	119.96 (14)	O5—S2—O4	119.27 (17)
O1—S1—N1	109.45 (13)	O5—S2—N2	109.80 (14)
O2—S1—N1	104.02 (12)	O4—S2—N2	103.43 (13)
O1—S1—C1	108.08 (13)	O5—S2—C12	108.93 (16)
O2—S1—C1	108.55 (13)	O4—S2—C12	108.21 (14)
N1—S1—C1	105.93 (12)	N2—S2—C12	106.43 (13)
			
C6—C1—C2—C3	−0.7 (4)	C17—C12—C13—C14	0.2 (5)
S1—C1—C2—C3	−177.3 (3)	S2—C12—C13—C14	−178.4 (3)
C1—C2—C3—C4	0.1 (5)	C12—C13—C14—C15	−0.6 (6)
C2—C3—C4—C5	0.9 (5)	C13—C14—C15—C16	0.4 (7)
C3—C4—C5—C6	−1.3 (5)	C14—C15—C16—C17	0.2 (7)
C4—C5—C6—C1	0.6 (5)	C13—C12—C17—C16	0.4 (5)
C2—C1—C6—C5	0.4 (4)	S2—C12—C17—C16	179.0 (3)
S1—C1—C6—C5	177.0 (2)	C15—C16—C17—C12	−0.6 (6)
O3—C7—C8—C9	−7.8 (4)	O6—C18—C19—C20	−2.7 (5)
N1—C7—C8—C9	172.8 (3)	N2—C18—C19—C20	178.1 (3)
O3—C7—C8—C10	−128.7 (3)	O6—C18—C19—C22	−124.9 (4)
N1—C7—C8—C10	51.9 (4)	N2—C18—C19—C22	56.0 (4)
O3—C7—C8—C11	111.1 (3)	O6—C18—C19—C21	115.7 (4)
N1—C7—C8—C11	−68.3 (3)	N2—C18—C19—C21	−63.4 (4)
O3—C7—N1—S1	3.8 (4)	O6—C18—N2—S2	−2.9 (5)
C8—C7—N1—S1	−176.8 (2)	C19—C18—N2—S2	176.2 (2)
C7—N1—S1—O1	51.8 (3)	C18—N2—S2—O5	−53.0 (3)
C7—N1—S1—O2	−178.8 (2)	C18—N2—S2—O4	178.7 (3)
C7—N1—S1—C1	−64.5 (3)	C18—N2—S2—C12	64.7 (3)
C6—C1—S1—O1	−5.4 (3)	C13—C12—S2—O5	22.0 (3)
C2—C1—S1—O1	171.3 (2)	C17—C12—S2—O5	−156.6 (2)
C6—C1—S1—O2	−136.9 (2)	C13—C12—S2—O4	153.0 (2)
C2—C1—S1—O2	39.7 (3)	C17—C12—S2—O4	−25.5 (3)
C6—C1—S1—N1	111.9 (2)	C13—C12—S2—N2	−96.4 (2)
C2—C1—S1—N1	−71.5 (3)	C17—C12—S2—N2	85.1 (2)

### Hydrogen-bond geometry (Å, °)

**Table:**

*D*—H···*A*	*D*—H	H···*A*	*D*···*A*	*D*—H···*A*
N1—H1N···O4^i^	0.86	2.09	2.946 (3)	171
N2—H2N···O2^ii^	0.86	2.32	3.094 (3)	151

Symmetry codes: (i) *x*, *y*+1, *z*; (ii) *x*, *y*−1, *z*.
